# Supplementary material for: Air pollution and individuals’ mental well-being in the adult population in United Kingdom: A spatial-temporal longitudinal study and the moderating effect of ethnicity
Source: PLoS One. 2022 Mar 9;17(3):e0264394. doi: 10.1371/journal.pone.0264394 (PMC8906596; doi:10.1371/journal.pone.0264394)
Supplement: S1 File — (DOCX) [file pone.0264394.s001.docx]

**Research Article Title:** Air pollution and individuals’ mental well-being in the adult population in United Kingdom: A spatial-temporal longitudinal study and the moderating effect of ethnicity

**Supporting Information (S1):** Additional analysis and sensitivity check-ups

Table 1: The association of individuals’ reported mental wellbeing with the socio-demographic and lifestyle covariates (N=349,748 surveys from 60,146 individuals)

|  | | **Mental well-being** **(GHQ0-36^a^ ≥ 12)** | **Mental well-being (GHQ0-12^b^ ≥ 2)** | **Mental well-being (GHQ0-12^b^ ≥ 4)** |
| --- | --- | --- | --- | --- |
|  |  | OR [95%CI] | OR [95%CI] | OR [95%CI] |
| **Ethnicity** | British white | Ref | Ref | Ref |
|  | Other white | 1.02 [0.93, 1.11] | 1.00 [0.92, 1.09] | 0.98 [0.90, 1.08] |
|  | Indian | 1.06 [0.95, 1.17] | 0.99 [0.89, 1.09] | 1.02 [0.91, 1.13] |
|  | Pakistani/Bangladeshi | 1.17 [1.06, 1.29]** | 1.09 [1.00, 1.19] | 1.13 [1.02, 1.25]* |
|  | Black/African/Caribbean | 0.65 [0.59, 0.71]** | 0.86 [0.79, 0.94]** | 0.81 [0.73, 0.90]** |
|  | Mixed ethnicities | 1.15 [1.01, 1.32]* | 1.21 [1.08, 1.37]** | 1.26 [1.10, 1.43]** |
|  | Other ethnicities | 1.12 [1.01, 1.24]* | 1.03 [0.94, 1.14] | 1.00 [0.89, 1.11] |
| **Country of birth** | Born in the UK | Ref | Ref | Ref |
|  | Not born in the UK | 0.83 [0.78, 0.89]** | 0.91 [0.85, 0.97]** | 0.85 [0.79, 0.91]** |
|  | No answer | 1.01 [0.95, 1.07] | 0.98 [0.93, 1.04] | 1.04 [0.98, 1.10] |
| **Age** | 16-18 | Ref | Ref | Ref |
|  | 19-23 | 1.24 [1.16, 1.32]** | 0.99 [0.93, 1.06] | 1.04 [0.97, 1.12] |
|  | 24-28 | 1.45 [1.33, 1.57]** | 0.97 [0.90, 1.05] | 1.07 [0.98, 1.18] |
|  | 29-33 | 1.54 [1.41, 1.69]** | 0.96 [0.88, 1.04] | 1.07 [0.97, 1.18] |
|  | 34-38 | 1.55 [1.42, 1.70]** | 0.91 [0.83, 0.99]* | 1.07 [0.97, 1.18] |
|  | 39-43 | 1.61 [1.47, 1.77]** | 0.87 [0.80, 0.95]** | 1.10 [1.00, 1.22] |
|  | 44-48 | 1.67 [1.53, 1.83]** | 0.88 [0.81, 0.96]** | 1.17 [1.06, 1.29]** |
|  | 49-53 | 1.74 [1.59, 1.91]** | 0.91 [0.83, 0.99]* | 1.20 [1.09, 1.33]** |
|  | 54-58 | 1.53 [1.39, 1.68]** | 0.79 [0.73, 0.87]** | 1.03 [0.93, 1.14] |
|  | 59-63 | 1.10 [1.00, 1.21] | 0.60 [0.55, 0.66]** | 0.76 [0.68, 0.84]** |
|  | 64-68 | 0.71 [0.64, 0.78]** | 0.40 [0.36, 0.44]** | 0.49 [0.44, 0.54]** |
|  | 69-73 | 0.62 [0.56, 0.68]** | 0.38 [0.34, 0.42]** | 0.41 [0.36, 0.46]** |
|  | 74-78 | 0.69 [0.62, 0.77]** | 0.45 [0.40, 0.49]** | 0.46 [0.41, 0.52]** |
|  | >78 | 0.78 [0.70, 0.88]** | 0.59 [0.53, 0.66]** | 0.56 [0.49, 0.64]** |
| **Gender** | Male | Ref | Ref | Ref |
|  | Female | 1.72 [1.66, 1.78]** | 1.62 [1.56, 1.67]** | 1.69 [1.62, 1.75]** |
| **Education** | University degree | Ref | Ref | Ref |
|  | High school degree | 1.04 [0.99, 1.08] | 0.96 [0.92, 1.00] | 0.98 [0.93, 1.03] |
|  | Lower educational levels | 1.14 [0.97, 1.34] | 0.98 [0.84, 1.14] | 0.99 [0.83, 1.18] |
|  | Other qualifications | 1.17 [1.12, 1.24]** | 1.08 [1.03, 1.13]** | 1.07 [1.02, 1.13]* |
|  | Still a student | 0.85 [0.79, 0.91]** | 0.90 [0.84, 0.96]** | 0.85 [0.78, 0.91]** |
| **Marital status** | Married | Ref | Ref | Ref |
|  | Living as a couple | 1.13 [1.07, 1.18]** | 1.17 [1.12, 1.23]** | 1.21 [1.15, 1.27]** |
|  | Widowed | 1.32 [1.23, 1.42]** | 1.33 [1.24, 1.42]** | 1.45 [1.34, 1.57]** |
|  | Divorced/separated | 1.48 [1.40, 1.57]** | 1.52 [1.44, 1.60]** | 1.60 [1.50, 1.69]** |
|  | Single never married | 1.38 [1.31, 1.45]** | 1.31 [1.25, 1.38]** | 1.39 [1.32, 1.47]** |
|  | No answer | 1.27 [1.01, 1.59]* | 1.30 [1.04, 1.63]* | 1.32 [1.03, 1.71]* |
| **Occupation** | Managers/Professionals/employers | Ref | Ref | Ref |
|  | Non manual workers | 1.02 [0.98, 1.07] | 0.96 [0.91, 1.00] | 0.97 [0.92, 1.03] |
|  | Manual workers | 0.83 [0.79, 0.87]** | 0.77 [0.73, 0.81]** | 0.74 [0.69, 0.78]** |
|  | Not applicable: Student/ retired/Not working | 1.64 [1.56, 1.72]** | 1.57 [1.50, 1.65]** | 1.70 [1.61, 1.80]** |
|  | No answer | 1.15 [1.02, 1.29]* | 0.97 [0.86, 1.09] | 1.00 [0.87, 1.15] |
| **Subjective financial situation** | Living comfortably/doing alright | Ref | Ref | Ref |
|  | Living difficultly | 2.43 [2.37, 2.49]** | 2.31 [2.26, 2.37]** | 2.59 [2.52, 2.66]** |
|  | No answer | 1.61 [1.25, 2.07]** | 1.52 [1.19, 1.94]** | 1.67 [1.26, 2.21]** |
| **Smoking status** | Non-smoker | Ref | Ref | Ref |
|  | Smoker | 1.31 [1.27, 1.36]** | 1.27 [1.22, 1.31]** | 1.34 [1.29, 1.40]** |
|  | No answer | 1.19 [1.12, 1.25]** | 1.17 [1.11, 1.24]** | 1.10 [1.03, 1.18]** |
| **Time dummies** | 2009 | Ref | Ref | Ref |
|  | 2010 | 1.10 [1.05, 1.16]** | 0.96 [0.91, 1.01] | 1.00 [0.94, 1.06] |
|  | 2011 | 1.02 [0.97, 1.08] | 0.95 [0.90, 1.00] | 1.02 [0.96, 1.08] |
|  | 2012 | 1.00 [0.95, 1.06] | 0.96 [0.91, 1.01] | 1.04 [0.97, 1.10] |
|  | 2013 | 1.08 [1.03, 1.14]** | 1.02 [0.97, 1.07] | 1.13 [1.06, 1.20]** |
|  | 2014 | 1.05 [1.00, 1.11] | 0.98 [0.93, 1.04] | 1.11 [1.04, 1.18]** |
|  | 2015 | 1.05 [1.00, 1.11] | 0.95 [0.90, 1.00] | 1.09 [1.02, 1.16]** |
|  | 2016 | 1.21 [1.15, 1.28]** | 1.00 [0.95, 1.05] | 1.18 [1.11, 1.26]** |
|  | 2017 | 1.37 [1.29, 1.44]** | 1.04 [0.99, 1.10] | 1.25 [1.17, 1.33]** |
|  | 2018 | 1.43 [1.35, 1.51]** | 1.06 [1.00, 1.12]* | 1.25 [1.17, 1.33]** |
|  | 2019 | 1.51 [1.41, 1.61]** | 1.08 [1.01, 1.15]* | 1.34 [1.24, 1.45]** |

**P-value <0.01; *P-value<0.05;

GHQ0-36^a^ : GHQ scale composed of 12 questions, each scored using a Likert format: 0-1-2-3 and summed up by adding all the items generating a scale ranging from 0 to 36; the 0-36 scale is dichotomised using a cut-off score of 12 based on relevant literature into good mental well-being (score<12) and poor mental well-being (score ≥ 12);

GHQ0-12^b^ : GHQ scale composed of 12 questions, each scored using a simple binary format: 0-0-1-1 and summed up by adding all the items generating a scale ranging from 0 to 12; the 0-12 scale is dichotomised using two cut-off scores of 2 and 4 based on relevant literature into good mental well-being (score<2 or score<4) and poor mental well-being (score ≥ 2 or score ≥ 4);

Table 2: The association of individuals’ mental well-being with each of NO_2_, SO_2_, PM10, and PM2.5 air pollutants linked at the LSOAs level in separate models for individuals recruited in wave 1 of the UKHLS survey (N=204,214 surveys from 31,258 individuals)

|  | **Mental well-being (GHQ0-36^a^ ≥ 12)** | | **Mental well-being (GHQ0-12^b^ ≥ 2)** | | **Mental well-being (GHQ0-12^b^ ≥ 4)** | |  |  |  |  |  |
| --- | --- | --- | --- | --- | --- | --- | --- | --- | --- | --- | --- |
|  | **Model 1** | **Model 2** | **Model 1** | **Model 2** | **Model 1** | **Model 2** |  |  |  |  |  |
|  | OR [95%CI] | OR [95%CI] | OR [95%CI] | OR [95%CI] | OR [95%CI] | OR [95%CI] |  |  |  |  |  |
| **Overall pollution effect** | | | | | | |  |  |  |  |  |
| NO_2_ (µg/m^3^) | 1.14 [1.10, 1.18]** | 1.10 [1.06, 1.14]** | 1.19 [1.16, 1.23]** | 1.12 [1.09, 1.16]** | 1.18 [1.14, 1.23]** | 1.11 [1.07, 1.15]** |  |  |  |  |  |
| SO_2_ (µg/m^3^) | 1.48 [1.29, 1.69]** | 1.33 [1.16, 1.52]** | 1.44 [1.27, 1.65]** | 1.29 [1.13, 1.47]** | 1.51 [1.30, 1.76]** | 1.31 [1.12, 1.52]** |  |  |  |  |  |
| PM10 (µg/m^3^) | 1.26 [1.16, 1.37]** | 1.24 [1.14, 1.35]** | 1.37 [1.27, 1.49]** | 1.26 [1.17, 1.37]** | 1.30 [1.18, 1.42]** | 1.21 [1.10, 1.32]** |  |  |  |  |  |
| PM2.5 (µg/m^3^) | 1.39 [1.25, 1.56]** | 1.35 [1.21, 1.51]** | 1.57 [1.42, 1.75]** | 1.40 [1.26, 1.55]** | 1.46 [1.29, 1.65]** | 1.31 [1.16, 1.48]** |  |  |  |  |  |
| **Between pollution effect** | | | | | | |  |  |  |  | **Between pollution effect** |
| NO_2_ (µg/m^3^) | 1.14 [1.10, 1.19]** | 1.11 [1.06, 1.15]** | 1.20 [1.16, 1.25]** | 1.13 [1.08, 1.17]** | 1.18 [1.13, 1.23]** | 1.10 [1.05, 1.15]** |  |  |  |  |  |
| SO_2_ (µg/m^3^) | 4.17 [3.04, 5.70]** | 2.20 [1.64, 2.96]** | 2.76 [2.06, 3.71]** | 1.54 [1.17, 2.04]** | 3.44 [2.47, 4.81]** | 1.69 [1.24, 2.31]** |  |  |  |  |  |
| PM10 (µg/m^3^) | 1.20 [1.10, 1.32]** | 1.20 [1.09, 1.31]** | 1.36 [1.25, 1.49]** | 1.25 [1.15, 1.36]** | 1.28 [1.16, 1.41]** | 1.15 [1.03, 1.27]* |  |  |  |  |  |
| PM2.5 (µg/m^3^) | 1.34 [1.17, 1.52]** | 1.32 [1.16, 1.50]** | 1.60 [1.42, 1.80]** | 1.41 [1.25, 1.59]** | 1.45 [1.27, 1.67]** | 1.30 [1.14, 1.50]** |  |  |  |  |  |
| **Within pollution effect** | | | | | | |  |  |  |  | **Within pollution effect** |
| NO_2_ (µg/m^3^) | 1.01 [0.89, 1.16] | 0.98 [0.86, 1.12] | 1.03 [0.90, 1.18] | 1.00 [0.88, 1.14] | 1.08 [0.92, 1.26] | 1.05 [0.90, 1.23] |  |  |  |  |  |
| SO_2_ (µg/m^3^) | 0.94 [0.75, 1.17] | 1.01 [0.81, 1.26] | 0.98 [0.79, 1.23] | 1.09 [0.87, 1.36] | 0.96 [0.74, 1.24] | 1.09 [0.84, 1.40] |  |  |  |  |  |
| PM10 (µg/m^3^) | 1.20 [0.94, 1.51] | 1.11 [0.88, 1.41] | 1.24 [0.98, 1.58] | 1.13 [0.90, 1.43] | 1.52 [1.15, 2.00]** | 1.39 [1.02, 1.88] |  |  |  |  |  |
| PM2.5 (µg/m^3^) | 1.19 [0.90, 1.59] | 1.11 [0.84, 1.47] | 1.35 [1.02, 1.79]* | 1.21 [0.91, 1.61] | 1.54 [1.11, 2.15]* | 1.38 [0.99, 1.91] |  |  |  |  |  |

**P-value <0.01; *P-value<0.05;

ORs and 95%CIs are expressed in terms of 10 µg/m^3^ increase in the air pollutants.

GHQ0-36^a^ : GHQ scale composed of 12 questions, each scored using a Likert format: 0-1-2-3 and summed up by adding all the items generating a scale ranging from 0 to 36; the 0-36 scale is dichotomised using a cut-off score of 12 based on relevant literature into good mental well-being (score<12) and poor mental well-being (score ≥ 12);

GHQ0-12^b^ : GHQ scale composed of 12 questions, each scored using a simple binary format: 0-0-1-1 and summed up by adding all the items generating a scale ranging from 0 to 12; the 0-12 scale is dichotomised using two cut-off scores of 2 and 4 based on relevant literature into good mental well-being (score<2 or score<4) and poor mental well-being (score ≥ 2 or score ≥ 4);

Model 1 is adjusted for age, gender and year dummies (2009-2019); Model 2 is adjusted for age, gender, ethnicity, country of birth, marital status, education, occupation, perceived financial situation, smoking status and year dummies (2009-2019).

Table 3: The association of individuals’ mental well-being with each of NO_2_, SO_2_, PM10, and PM2.5 air pollutants linked at the local authority level in separate models for individuals recruited in wave 1 of the UKHLS survey (N=204,214 surveys from 31,258 individuals)

|  | **Mental well-being (GHQ0-36^a^ ≥ 12)** | | **Mental well-being (GHQ0-12^b^ ≥ 2)** | | **Mental well-being (GHQ0-12^b^ ≥ 4)** | |  |  |  |  |  |
| --- | --- | --- | --- | --- | --- | --- | --- | --- | --- | --- | --- |
|  | **Model 1** | **Model 2** | **Model 1** | **Model 2** | **Model 1** | **Model 2** |  |  |  |  |  |
|  | OR [95%CI] | OR [95%CI] | OR [95%CI] | OR [95%CI] | OR [95%CI] | OR [95%CI] |  |  |  |  |  |
| **Overall pollution effect** | | | | | | |  |  |  |  |  |
| NO_2_ (µg/m^3^) | 1.13 [1.08, 1.18]** | 1.10 [1.06, 1.15]** | 1.17 [1.13, 1.22]** | 1.12 [1.08, 1.17]** | 1.15 [1.10, 1.20]** | 1.10 [1.05, 1.14]** |  |  |  |  |  |
| SO_2_ (µg/m^3^) | 1.70 [1.38, 2.10]** | 1.57 [1.28, 1.92]** | 1.47 [1.19, 1.81]** | 1.35 [1.11, 1.65]** | 1.41 [1.11, 1.80]** | 1.28 [1.02, 1.60]* |  |  |  |  |  |
| PM10 (µg/m^3^) | 1.20 [1.08, 1.33]** | 1.19 [1.09, 1.31]** | 1.26 [1.14, 1.38]** | 1.21 [1.11, 1.32]** | 1.17 [1.06, 1.31]** | 1.14 [1.04, 1.26]** |  |  |  |  |  |
| PM2.5 (µg/m^3^) | 1.31 [1.14, 1.50]** | 1.30 [1.14, 1.47]** | 1.42 [1.25, 1.61]** | 1.33 [1.18, 1.50]** | 1.28 [1.11, 1.48]** | 1.22 [1.07, 1.40]** |  |  |  |  |  |
| **Between pollution effect** | | | | | | |  |  |  |  | **Between pollution effect** |
| NO_2_ (µg/m^3^) | 1.11 [1.06, 1.17]** | 1.09 [1.04, 1.14]** | 1.18 [1.13, 1.23]** | 1.12 [1.07, 1.17]** | 1.14 [1.08, 1.20]** | 1.08 [1.03, 1.14]** |  |  |  |  |  |
| SO_2_ (µg/m^3^) | 4.29 [2.68, 6.88]** | 2.61 [1.73, 3.96]** | 3.82 [2.44, 5.97]** | 2.30 [1.55, 3.42]** | 4.06 [2.50, 6.60]** | 2.26 [1.47, 3.47]** |  |  |  |  |  |
| PM10 (µg/m^3^) | 1.13 [1.004, 1.27]* | 1.16 [1.05, 1.28]** | 1.26 [1.14, 1.40]** | 1.22 [1.11, 1.34]** | 1.17 [1.04, 1.32]** | 1.15 [1.03, 1.27]* |  |  |  |  |  |
| PM2.5 (µg/m^3^) | 1.22 [1.03, 1.43]* | 1.26 [1.09, 1.45]** | 1.44 [1.25, 1.68]** | 1.36 [1.19, 1.56]** | 1.29 [1.09, 1.52]** | 1.24 [1.07, 1.44]** |  |  |  |  |  |
| **Within pollution effect** | | | | | | |  |  |  |  | **Within pollution effect** |
| NO_2_ (µg/m^3^) | 1.11 [0.94, 1.30] | 1.10 [0.94, 1.30] | 1.09 [0.92, 1.28] | 1.08 [0.92, 1.27] | 1.08 [0.89, 1.31] | 1.08 [0.89, 1.30] |  |  |  |  |  |
| SO_2_ (µg/m^3^) | 1.08 [0.76, 1.52] | 1.10 [0.78, 1.55] | 0.99 [0.70, 1.39] | 1.03 [0.73, 1.45] | 0.87 [0.58, 1.31] | 0.92 [0.62, 1.38] |  |  |  |  |  |
| PM10 (µg/m^3^) | 1.22 [0.94, 1.58] | 1.18 [0.91, 1.53] | 1.17 [0.90, 1.52] | 1.12 [0.86, 1.45] | 1.45 [1.07, 1.96]* | 1.39 [1.02, 1.88] |  |  |  |  |  |
| PM2.5 (µg/m^3^) | 1.14 [0.83, 1.57] | 1.11 [0.81, 1.53] | 1.22 [0.88, 1.68] | 1.15 [0.84, 1.59] | 1.34 [0.92, 1.95] | 1.26 [0.87, 1.82] |  |  |  |  |  |

**P-value <0.01; *P-value<0.05;

ORs and 95%CIs are expressed in terms of 10 µg/m^3^ increase in the air pollutants.

GHQ0-36^a^ : GHQ scale composed of 12 questions, each scored using a Likert format: 0-1-2-3 and summed up by adding all the items generating a scale ranging from 0 to 36; the 0-36 scale is dichotomised using a cut-off score of 12 based on relevant literature into good mental well-being (score<12) and poor mental well-being (score ≥ 12);

GHQ0-12^b^ : GHQ scale composed of 12 questions, each scored using a simple binary format: 0-0-1-1 and summed up by adding all the items generating a scale ranging from 0 to 12; the 0-12 scale is dichotomised using two cut-off scores of 2 and 4 based on relevant literature into good mental well-being (score<2 or score<4) and poor mental well-being (score ≥ 2 or score ≥ 4);

Model 1 is adjusted for age, gender and year dummies (2009-2019); Model 2 is adjusted for age, gender, ethnicity, country of birth, marital status, education, occupation, perceived financial situation, smoking status and year dummies (2009-2019).

Fig 1: The overall effect of air pollution linked at the LSOAs level on individuals’ reported mental wellbeing by ethnicity and country of birth for individuals recruited in wave 1 of the UKHLS survey (N=204,214 surveys from 31,258 individuals)


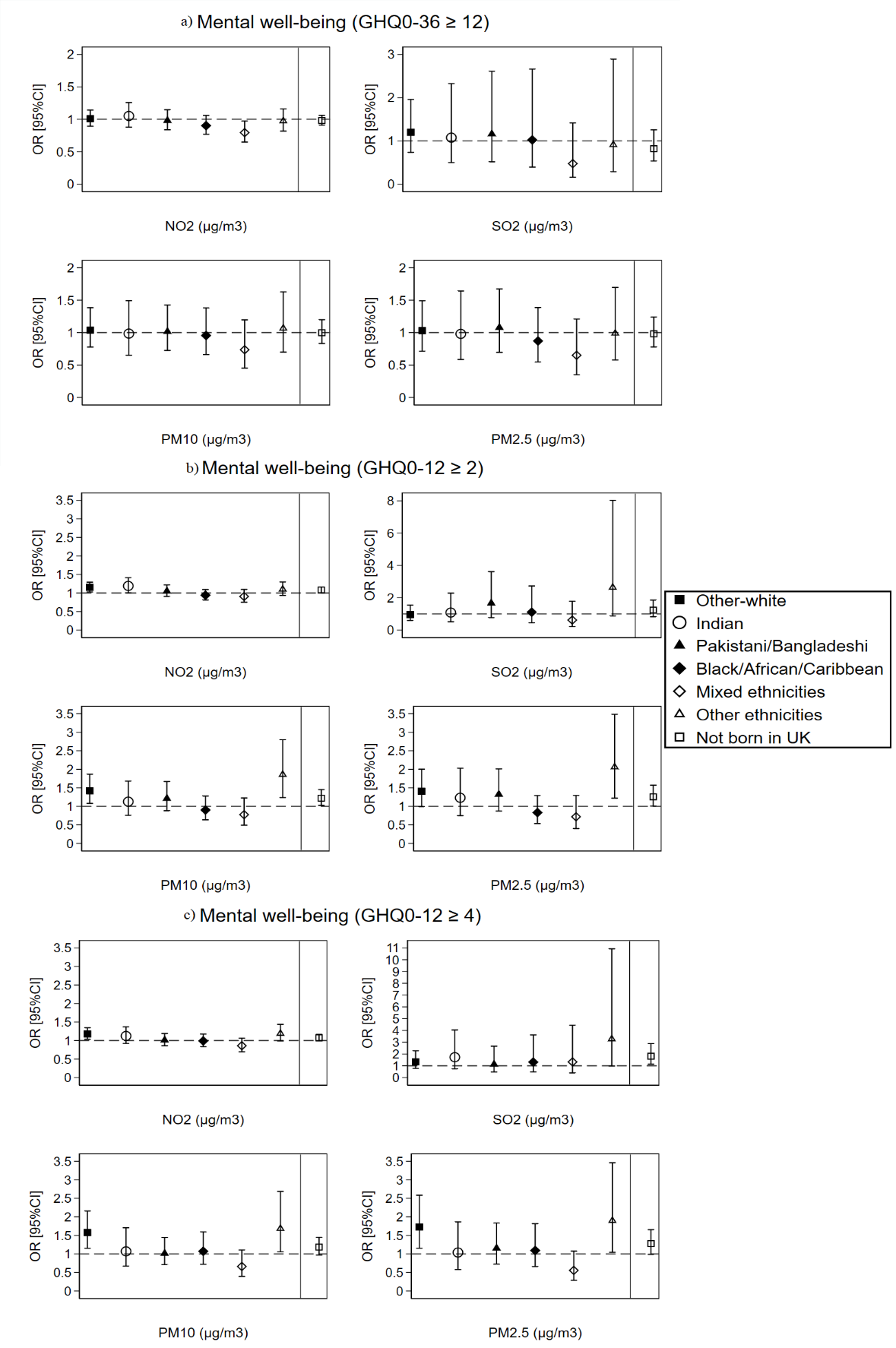


The dashed line is placed at OR=1 as a cut-off for statistically insignificant results; The solid line separates between the air pollution-ethnicity interaction models and the air pollution-country of birth interaction models; Air pollution-ethnicity interaction models are adjusted for country of birth, age, gender, marital status, education, occupation, subjective financial situation, smoking status, and year dummies (2009 to 2019); Air pollution-country of birth interaction models are adjusted for ethnicity, age, gender, marital status, education, occupation, subjective financial situation, smoking status, and year dummies (2009 to 2019).

Fig 2: The overall effect of air pollution linked at the local authority level on individuals’ reported mental wellbeing by ethnicity and country of birth for individuals recruited in wave 1 of the UKHLS survey (N=204,214 surveys from 31,258 individuals)


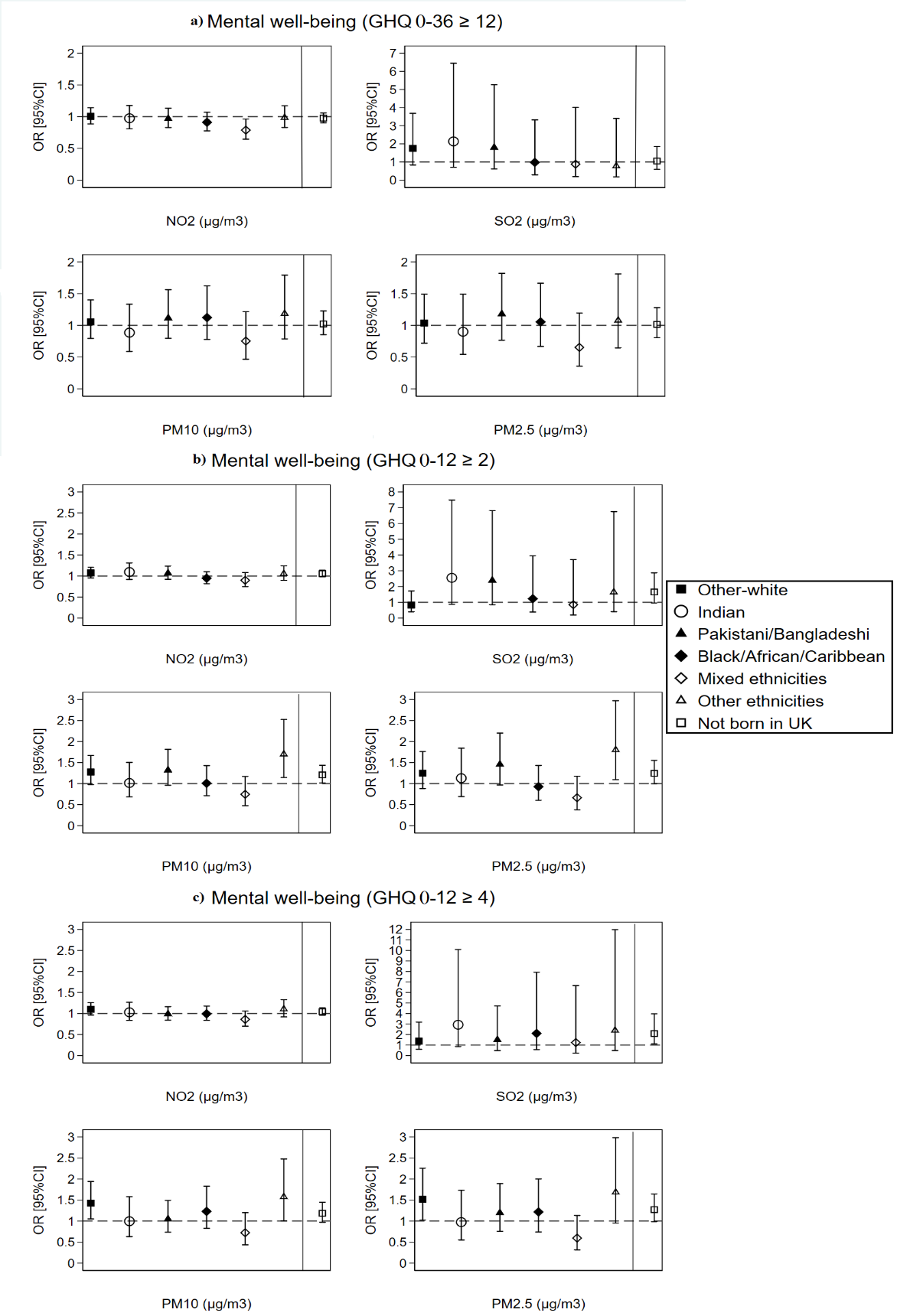


The dashed line is placed at OR=1 as a cut-off for statistically insignificant results; The solid line separates between the air pollution-ethnicity interaction models and the air pollution-country of birth interaction models; Air pollution-ethnicity interaction models are adjusted for country of birth, age, gender, marital status, education, occupation, subjective financial situation, smoking status, and year dummies (2009 to 2019); Air pollution-country of birth interaction models are adjusted for ethnicity, age, gender, marital status, education, occupation, subjective financial situation, smoking status, and year dummies (2009 to 2019).

Fig 3: The *between* (spatial) effect of air pollution linked at the LSOAs level on individuals’ mental well-being by ethnicity and country of birth (N=349,748 surveys from 60,146 individuals)


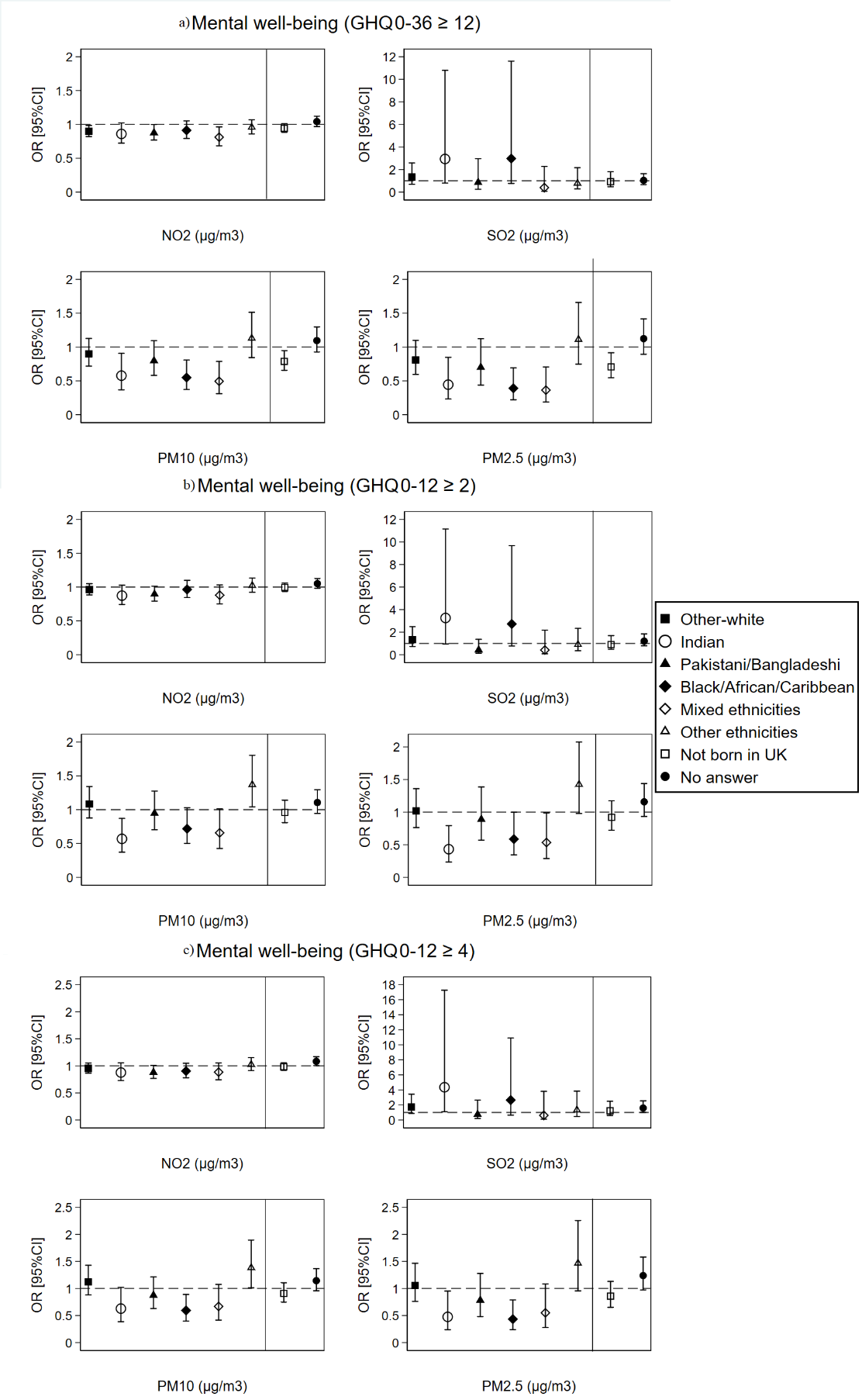


The dashed line is placed at OR=1 as a cut-off for statistically insignificant results; The solid line separates between the air pollution-ethnicity interaction models and the air pollution-country of birth interaction models; Air pollution-ethnicity interaction models are adjusted for country of birth, age, gender, marital status, education, occupation, perceived financial situation, smoking status, and year dummies (2009 to 2019); Air pollution-country of birth interaction models are adjusted for ethnicity, age, gender, marital status, education, occupation, perceived financial situation, smoking status, and year dummies (2009 to 2019).

Fig 4: The *within* (temporal) effect of air pollution linked at the LSOAs level on individuals’ mental well-being by ethnicity and country of birth (N=349,748 surveys from 60,146 individuals)


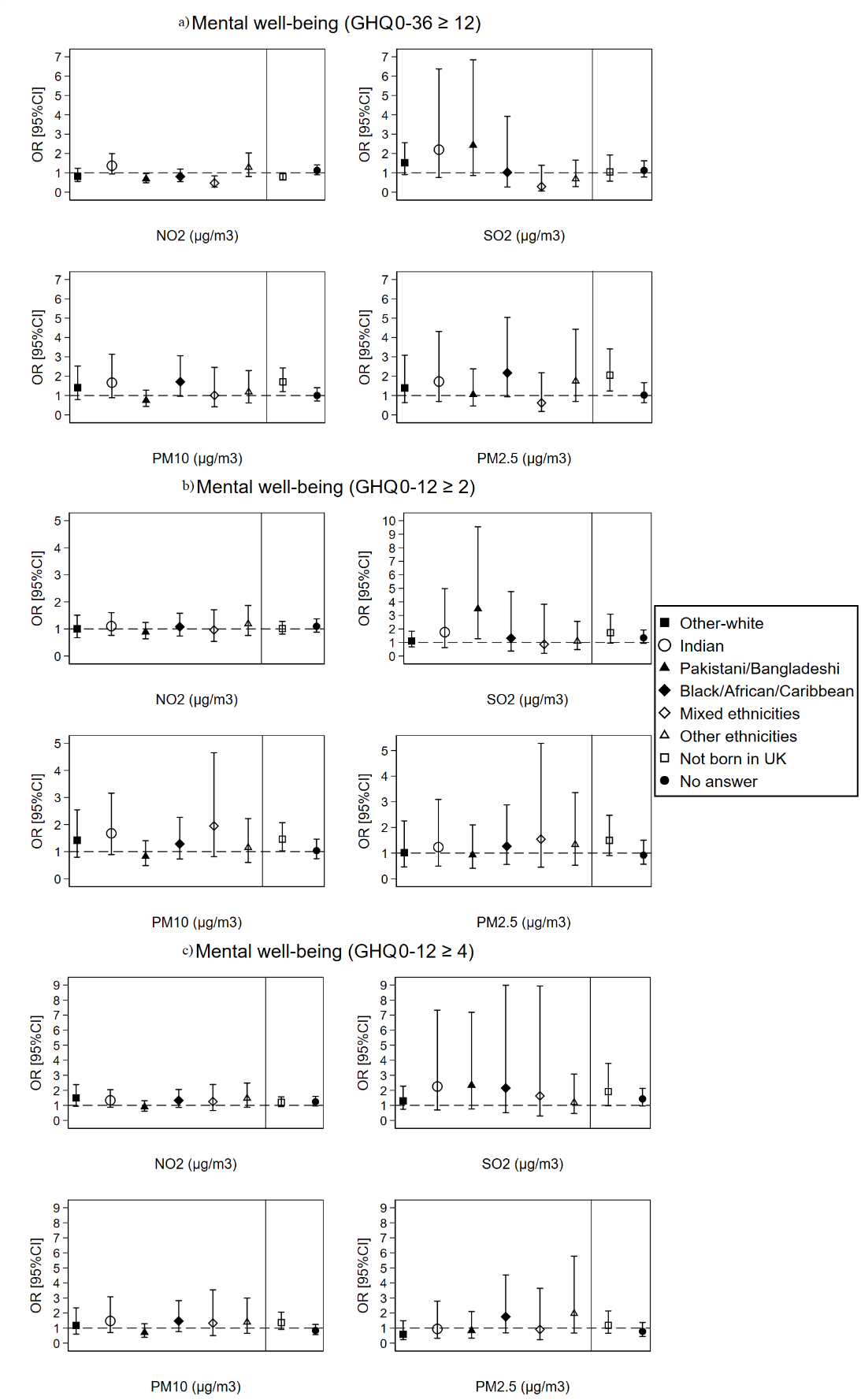


The dashed line is placed at OR=1 as a cut-off for statistically insignificant results; The solid line separates between the air pollution-ethnicity interaction models and the air pollution-country of birth interaction models; Air pollution-ethnicity interaction models are adjusted for country of birth, age, gender, marital status, education, occupation, perceived financial situation, smoking status, and year dummies (2009 to 2019); Air pollution-country of birth interaction models are adjusted for ethnicity, age, gender, marital status, education, occupation, perceived financial situation, smoking status, and year dummies (2009 to 2019).

Fig 5: The *between* (spatial) effect of air pollution linked at the local authority level on individuals’ mental well-being by ethnicity and country of birth (N=349,748 surveys from 60,146 individuals)


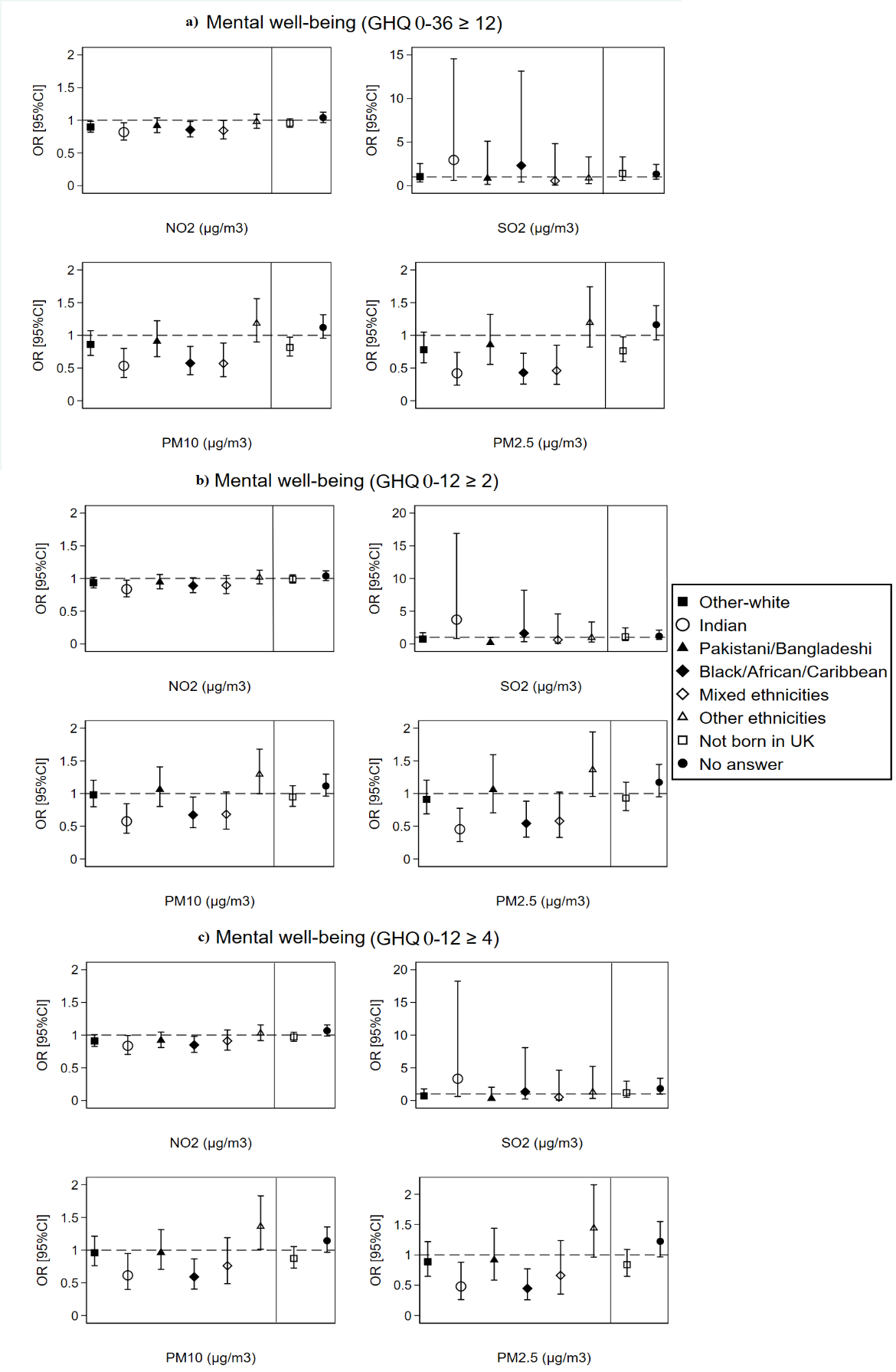


The dashed line is placed at OR=1 as a cut-off for statistically insignificant results; The solid line separates between the air pollution-ethnicity interaction models and the air pollution-country of birth interaction models; Air pollution-ethnicity interaction models are adjusted for country of birth, age, gender, marital status, education, occupation, perceived financial situation, smoking status, and year dummies (2009 to 2019); Air pollution-country of birth interaction models are adjusted for ethnicity, age, gender, marital status, education, occupation, perceived financial situation, smoking status, and year dummies (2009 to 2019).

Fig 6: The *within* (temporal) effect of air pollution linked at the local authority level on individuals’ mental well-being by ethnicity and country of birth (N=349,748 surveys from 60,146 individuals)


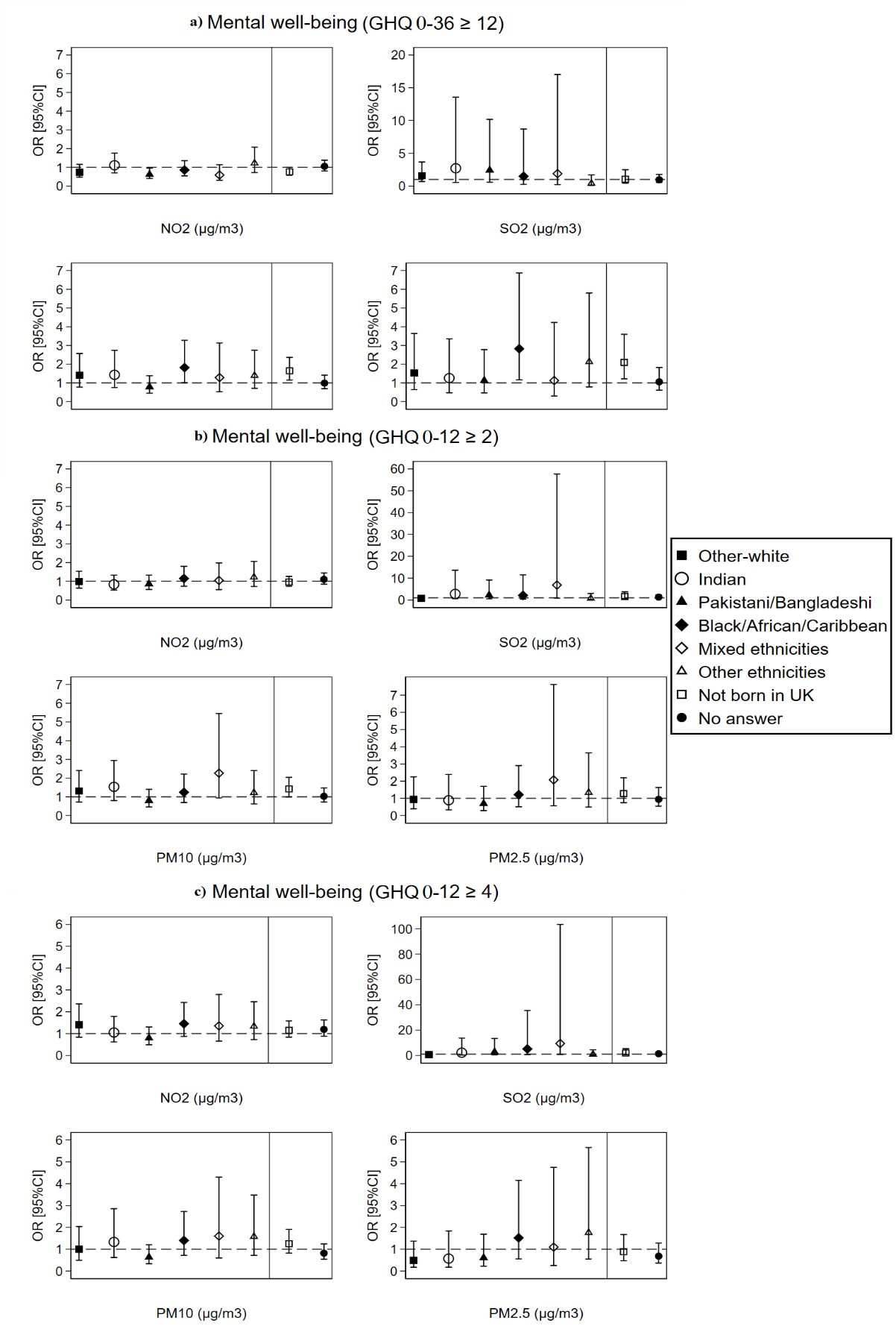


The dashed line is placed at OR=1 as a cut-off for statistically insignificant results; The solid line separates between the air pollution-ethnicity interaction models and the air pollution-country of birth interaction models; Air pollution-ethnicity interaction models are adjusted for country of birth, age, gender, marital status, education, occupation, perceived financial situation, smoking status, and year dummies (2009 to 2019); Air pollution-country of birth interaction models are adjusted for ethnicity, age, gender, marital status, education, occupation, perceived financial situation, smoking status, and year dummies (2009 to 2019).

Table 4: Description of the area of residence (urban or rural) for ethnic groups and country of birth in the UKHLS survey using Chi2 test tabulation (N=349,748 surveys from 60,146 individuals)

|  | | Urban area | Rural area | Total |
| --- | --- | --- | --- | --- |
| **Ethnicity (Chi2 test P-value=0.000)** | | | | |
| British-white | Count | 199,177 | 79,747 | 278,924 |
|  | % | 71.41 | 28.59 | 100 |
| Other-white | Count | 12,294 | 5,017 | 17,311 |
|  | % | 71.02 | 28.98 | 100 |
| Indian | Count | 11,028 | 201 | 11,229 |
|  | % | 98.21 | 1.79 | 100 |
| Pakistani/Bangladeshi | Count | 13,224 | 68 | 13,292 |
|  | % | 99.49 | 0.51 | 100 |
| Black/African/Caribbean | Count | 12,628 | 143 | 12,771 |
|  | % | 98.88 | 1.12 | 100 |
| mixed ethnicities | Count | 5,412 | 353 | 5,765 |
|  | % | 93.88 | 6.12 | 100 |
| other ethnicities | Count | 9,115 | 1,341 | 10,456 |
|  | % | 87.17 | 12.83 | 100 |
| Total | Count | 262,878 | 86,870 | 349,748 |
|  | % | 75.16 | 24.84 | 100 |
| **Country of birth (Chi2 test P-value=0.000)** | | | | |
| Born in the UK | Count | 180,409 | 61,633 | 242,042 |
|  | % | 74.54 | 25.46 | 100 |
| Not born in the UK | Count | 39,452 | 3,200 | 42,652 |
|  | % | 92.50 | 7.50 | 100 |
| No answer | Count | 43,017 | 22,037 | 65,054 |
|  | % | 66.13 | 33.87 | 100 |
| Total | Count | 262,878 | 86,870 | 349,748 |
|  | % | 75.16 | 24.84 | 100 |
